# Precise automated determination of the total and segmented right ventricular volumes for functional studies of the right ventricle using CMR

**DOI:** 10.1186/1532-429X-13-S1-P223

**Published:** 2011-02-02

**Authors:** Dominik Gabbert, Andreas Entenmann, Michael Jerosch-Herold, Chris Hart, Inga Voges, Traudel Hansen, Hans Heiner Kramer, Carsten Rickers

**Affiliations:** 1 University Hospital Schleswig-Holstein, Dept. Ped. Card., Kiel, Germany; 2 Brigham and Womens Hospital, Harvard Medical School, Boston, MA, USA

## Background

Cardiac magnetic resonance (CMR) is an acknowledged standard for quantification of left ventricular volumes, but the acceptance of CMR to measure the volume of the right ventricle (RV), including sub-units, such as inlet, outlet, and apical segment has been limited. In patients with Fallot (TOF), the size and function of the right ventricular outflow tract (RVOT) in the course of an RR-interval can serve as indication for re-operation in cases of pulmonary stenosis, regurgitation, or RVOT aneurysms. in addition to the results of conductance examinations. However, none of the available software applications provides a suitable systematic subdivison of the total volume. A novel algorithm was developed for this purpose and tested in this study.

## Methods

We present an automated method for the determination of RV volumes based on short axis cine series from CMR examinations. The contour detection proceeds iteratively through slices and phases. Manual interaction is possible. For further functional analysis of the right ventricle, a subdivision into compartiments is applied. The subdivision is performed automatically from flat or curved layers as defined by landmarks identified by the investigator. The landmarks include the crista supraventricularis, the set in of trabeculary structures, and the apex. The conventional definitions of the compartiments are loose, therefore two definitions of the outlet are compared to each other: one robust and simple definition using flat separating layers, and a more complicated but flexible definition based on a curved layer.

## Results

Depending on the quality of the images, this contour detection is successful (i. e. requires no further manual interaction) in up to about 95% of images in the mid-ventricular region. Papillary structures in the apex as well as a weakly separable atrium in the basal region may require manual interactions. Volumes are provided for all phases in total and in specific regions. Stroke volume and ejection fraction arise naturally. The volumes obtained from the two outlet definitions turn out to agree typically within 10% of the total volume and thus do not lead to contradicting conclusions.

## Conclusion

The application of this fast iterative contour detection method and volumetry may allow the evaluation of the chamber volumes for the full heart cycle in clinical routine. The automatic volume segmentation provides useful information about the function of a particular segment such as the RV outlet in TOF patients where it can complete the indications for a surgery.

